# Supplementary material for: Hemopexin and α1-microglobulin heme scavengers with differential involvement in preeclampsia and fetal growth restriction
Source: PLoS One. 2020 Sep 11;15(9):e0239030. doi: 10.1371/journal.pone.0239030 (PMC7485876; doi:10.1371/journal.pone.0239030)

Hemopexin and α_1-microglobulin heme scavengers with differential involvement in preeclampsia and fetal growth restriction

Lina Youssef[1,2*], Lena Erlandsson[1], Bo Åkerström[3], Jezid Miranda[2], Cristina Paules[2], Francesca Crovetto[2,4], Fatima Crispi[2,4], Eduard Gratacos[2,4], Stefan R. Hansson[1,5].

[1] Section of Obstetrics and Gynecology, Department of Clinical Sciences Lund, Lund University, Sweden.

[2] BCNatal | Fetal Medicine Research Center (Hospital Clínic and Hospital Sant Joan de Déu), Institut d'Investigacions Biomèdiques August Pi i Sunyer (IDIBAPS), University of Barcelona, Barcelona, Spain.

[3] Section of infection Medicine, Department of Clinical Sciences, Lund University, Lund, Sweden.

[4] Centre for Biomedical Research on Rare Diseases (CIBER-ER), Madrid, Spain.

[5] Skåne University Hospital, Lund/Malmö, Sweden.

**Corresponding author:*

E-mail: lyoussef@clinic.cat

linayoussefdr@gmail.com

S1 Table. Concentrations of heme scavengers in maternal and fetal blood in the study population.

	Controls	FGR	PE	PE&FGR
Maternal blood concentrations				
Number of samples	49	47	45	51
Hemopexin (ug/ml)	1094 (±256)	1048 (±365)	857 (±305)*†	941 (±338)*†
A1M (ug/ml)	23.9 (±6.6)	24.9 (±8.1)	31.5 (±8.7)*†	33.4 (±8.4)*†
Cord blood concentrations				
Number of samples	45	42	40	37
Hemopexin (ug/ml)	250 (±113)	176 (±103)*†	211 (±96)	133 (±100)*†
A1M (ug/ml)	17.9 (±4.2)	15.6 (±4.1)*†	18.1 (±4.4)	15.6 (±3.8)*†

Data are mean (± standard deviation). A1M, α1-microglobulin; FGR, fetal growth restriction; PE, preeclampsia.

* $p<0.05$ by student *t* or Mann Whitney U tests as appropriate, compared to controls (unadjusted).

† $p<0.05$ by multiple regression compared to controls, adjusted for chronic hypertension, diabetes, assisted reproductive technologies, smoking, fetal sex and gestational age at sampling.

S2 Table. Concentrations of heme scavengers in maternal and fetal blood in preterm pregnancies.

	**Controls**	**FGR**	**PE**	**PE&FGR**
Maternal blood concentrations				
Number of samples	16	13	13	34
Hemopexin (ug/ml)	1120 (±289)	1083 (±321)	812 (±365)*†	913 (±322)*†
A1M (ug/ml)	21 (±5.6)	23.7 (±8.4)	31.7 (±9.6)*†	34.3 (±9)*†
Cord blood concentrations				
Number of samples	13	8	12	23
Hemopexin (ug/ml)	258 (±123)	174 (±154)*†	171 (±74)	99 (±81)*†
A1M (ug/ml)	19.7 (±4.8)	14.2 (±6)*†	17.6 (±4.6)	15.8 (±2.9)*†

Data are mean (± standard deviation). A1M, α_1-microglobulin; FGR, fetal growth restriction; PE, preeclampsia.

* $p<0.05$ by student *t* or Mann Whitney U tests as appropriate, compared to controls (unadjusted).

† $p<0.05$ by multiple regression compared to controls, adjusted for chronic hypertension, diabetes, assisted reproductive technologies, smoking and fetal sex.

S3 Table. Concentrations of heme scavengers in maternal and fetal blood in term pregnancies.

	Controls	FGR	PE	PE&FGR
Maternal blood concentrations				
Number of samples	33	34	32	17
Hemopexin (ug/ml)	1081 (±242)	1034 (±384)	875 (±282)*†	998 (±372)
A1M (ug/ml)	25.3 (±6.6)	25.4 (±8.1)	31.5 (±8.5)*	31.5 (±6.8)*
Cord blood concentrations				
Number of samples	32	34	28	14
Hemopexin (ug/ml)	247 (±111)	177 (±90.4)*	229 (±100)	189 (±105)
A1M (ug/ml)	17.1 (±3.7)	16 (±3.6)	18.4 (±4.4)	15.2 (±5)

Data are mean (± standard deviation). A1M, α_1-microglobulin; FGR, fetal growth restriction; PE, preeclampsia.

* $p<0.05$ by student *t* or Mann Whitney U tests as appropriate, compared to controls (unadjusted).

† $p<0.05$ by multiple regression compared to controls, adjusted for chronic hypertension, diabetes, assisted reproductive technologies, smoking and fetal sex.

S4 Table. Concentrations of heme scavengers in maternal and fetal blood in pregnancies with a male fetus.

	**Controls**	**FGR**	**PE**	**PE&FGR**
Maternal blood concentrations				
Number of samples	26	25	17	31
Hemopexin (ug/ml)	1115 (±277)	1074 (±411)	866 (±295)*†	956 (±343)
A1M (ug/ml)	23.9 (±7.3)	23.6 (±7.8)	29.6 (±8.9)*†	33.1 (±8.6)*†
Cord blood concentrations				
Number of samples	24	23	15	22
Hemopexin (ug/ml)	217 (±108)	162 (±105)	209 (±111)	113 (±101)*†
A1M (ug/ml)	17.2 (±4.4)	15.8 (±3.9)	19.1 (±5.1)	15.8 (±3.3)

Data are mean (± standard deviation). A1M, α_1-microglobulin; FGR, fetal growth restriction; PE, preeclampsia.

* $p<0.05$ by student *t* or Mann Whitney U tests as appropriate, compared to controls (unadjusted).

† $p<0.05$ by multiple regression compared to controls, adjusted for chronic hypertension, diabetes, assisted reproductive technologies, smoking and gestational age at delivery.

S5 Table. Concentrations of heme scavengers in maternal and fetal blood in pregnancies with a female fetus.

	**Controls**	**FGR**	**PE**	**PE&FGR**
Maternal blood concentrations				
Number of samples	23	22	28	20
Hemopexin (ug/ml)	1070 (±234)	1017 (±311)	852 (±317)*†	918 (±338)
A1M (ug/ml)	24 (±5.8)	26.4 (±8.4)	32.7 (±8.5)*†	33.8 (±8.2)*†
Cord blood concentrations				
Number of samples	21	19	25	15
Hemopexin (ug/ml)	288 (±109)	193 (±100)*†	213 (±88.3)*	162 (±92.7)*†
A1M (ug/ml)	18.7 (±3.9)	15.4 (±4.5)*†	17.6 (±3.9)	15.1 (±4.5)*†

Data are mean (± standard deviation). A1M, α_1-microglobulin; FGR, fetal growth restriction; PE, preeclampsia.

* $p<0.05$ by student *t* or Mann Whitney *U* tests as appropriate, compared to controls (unadjusted).

† $p<0.05$ by multiple regression compared to controls, adjusted for chronic hypertension, diabetes, assisted reproductive technologies, smoking and gestational age at sampling.

S1 Fig. An example of the standard curve used to quantify a) maternal and b) fetal α1-microglobulin concentrations in unknown plasma samples.

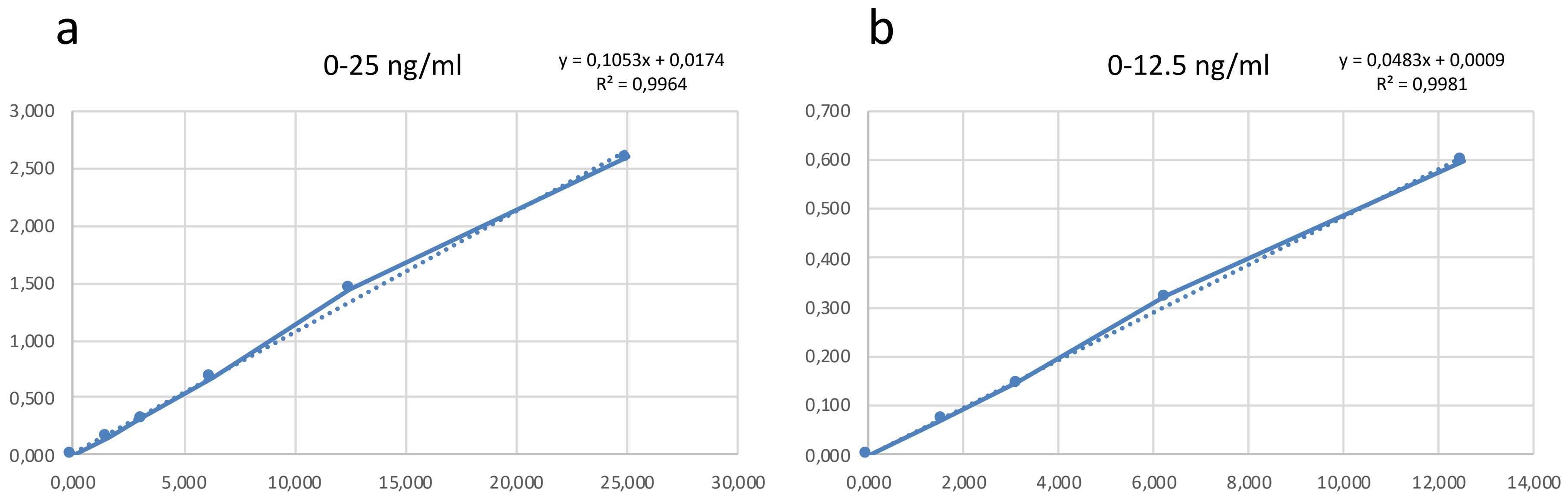

S2 Fig. Pairwise correlation between maternal blood a) hemopexin or b) α1-microglobulin and serum creatinine concentrations in preeclamptic pregnancies. Scatter and best fit line plots with Pearson correlation coefficient (r) and level of significance. A1M, α1-microglobulin.

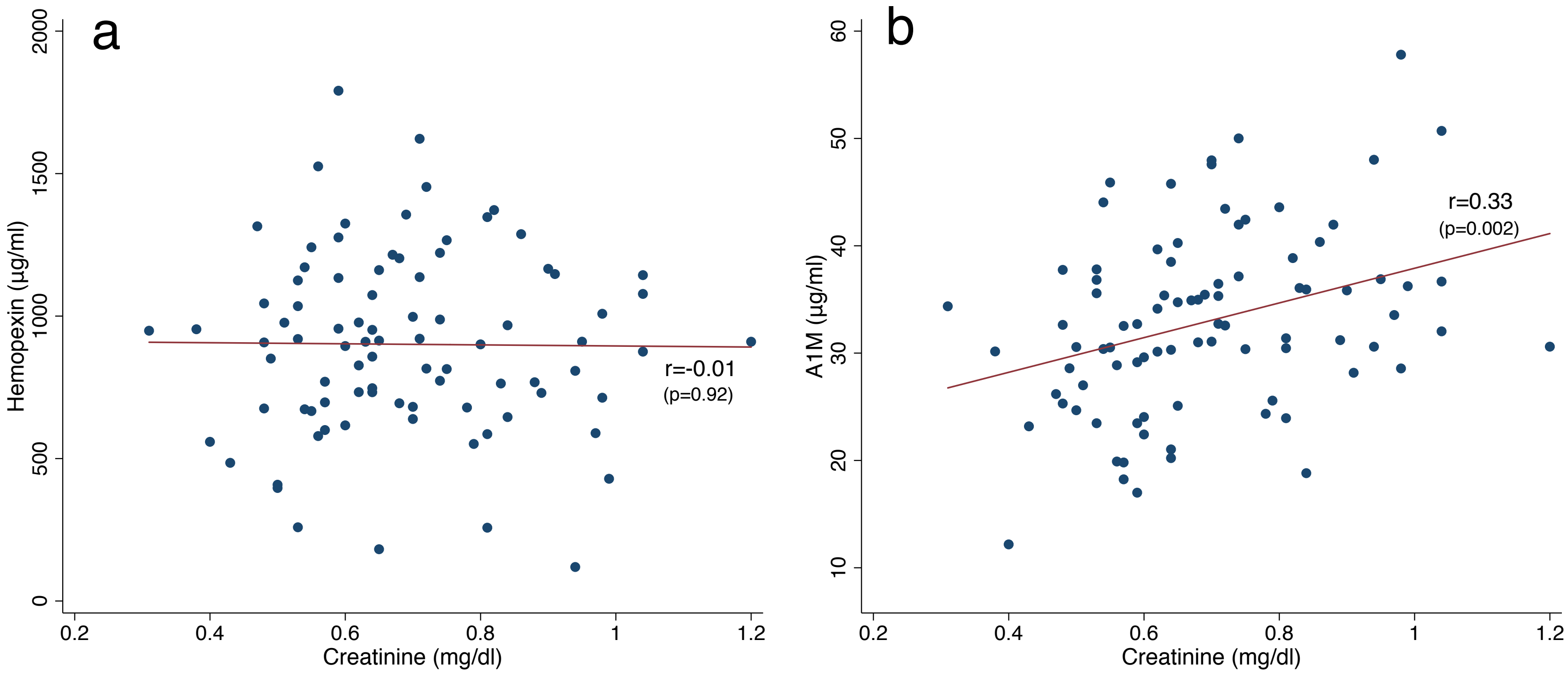

S3 Fig. a) Free fetal hemoglobin, b) LDH activity and c) erythropoietin cord blood concentrations in the study population. Boxes show median and interquartile range, whiskers represent 1.5 X interquartile range or the extremes of the distribution. FGR, fetal growth restriction; PE, preeclampsia; HbF, fetal hemoglobin; LDH, lactate dehydrogenase.

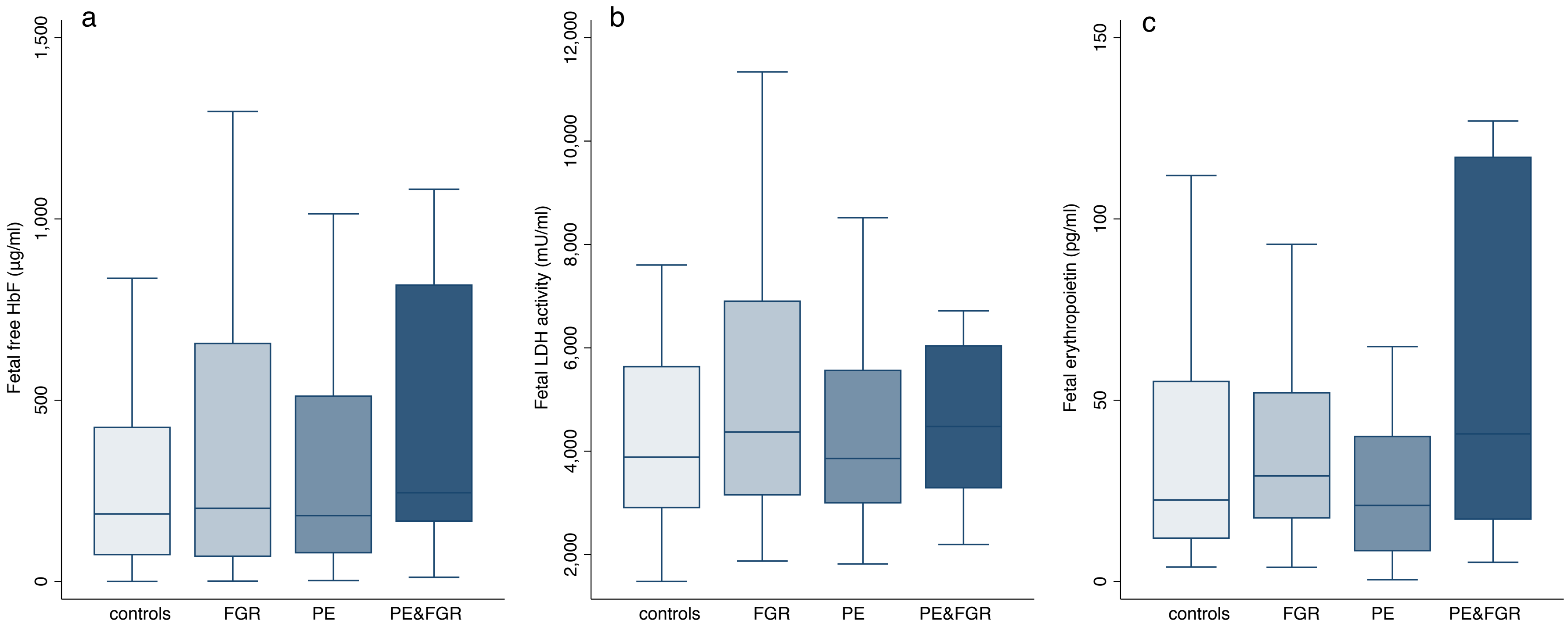

Supplement: S1 File — (PDF) [file pone.0239030.s001.pdf]
