# Supplementary material for: Hemopexin and α1-microglobulin heme scavengers with differential involvement in preeclampsia and fetal growth restriction
Source: PLoS One. 2020 Sep 11;15(9):e0239030. doi: 10.1371/journal.pone.0239030 (PMC7485876; doi:10.1371/journal.pone.0239030)
Supplement: Attachment — Submitted filename: 200709_Response_to_reviewers.docx [file pone.0239030.s002.docx]

Lina Youssef

Fetal medicine research center, BCNatal

Sabino de Arana 1, 08028 Barcelona, Spain

+34 932 275 400

lyoussef@clinic.cat

July 9, 2020

Dear Editor,

Thank you very much for considering our manuscript “Hemopexin and α1-microglobulin heme scavengers with differential involvement in preeclampsia and fetal growth restriction” (PONE-D-20-14717).

We would like to thank the reviewers for their detailed comments and suggestions for the manuscript. We believe that the comments have identified important areas which required improvement. Below, you will find a point by point description of how each comment was addressed in the manuscript.

**ACADEMIC EDITOR COMMENTS:**

There were two expert reviewers in the field that handled your manuscript. We thank them for their time. Although interest was found in your study, several major points arose during review that require your attention. Comments that need addressing include: 1) several questions about the methods, 2) the need for correlative studies, and 3) measures of oxidative stress. Please address all of the reviewers' comments in your revised manuscript.

**Answer: 1) the methods’ details requested by the reviewers have been added as specified below, 2) correlative studies have been added to the manuscript, 3) unfortunately, other biomarkers of oxidative stress have not been assessed in this study (this comment has been added to the limitations section).**

When submitting your revision, we need you to address these additional requirements.

1. Please ensure that your manuscript meets PLOS ONE's style requirements, including those for file naming. The PLOS ONE style templates can be found at

<https://journals.plos.org/plosone/s/file?id=wjVg/PLOSOne_formatting_sample_main_body.pdf> and

<https://journals.plos.org/plosone/s/file?id=ba62/PLOSOne_formatting_sample_title_authors_affiliations.pdf>

**Answer: the style and file names have been modified according to these instructions.**

2. We note that you have a patent relating to material pertinent to this article. Please provide an amended statement of Competing Interests to declare this patent (with details including name and number), along with any other relevant declarations relating to employment, consultancy, patents, products in development or modified products etc. Please confirm that this does not alter your adherence to all PLOS ONE policies on sharing data and materials, as detailed online in our guide for authors <http://journals.plos.org/plosone/s/competing-interests> by including the following statement: "This does not alter our adherence to PLOS ONE policies on sharing data and materials.” If there are restrictions on sharing of data and/or materials, please state these. Please note that we cannot proceed with consideration of your article until this information has been declared.

This information should be included in your cover letter; we will change the online submission form on your behalf.

**Answer: We added the patent details as follows. “SH and BÅ hold a patent related to the subject in this paper (PCT appl no. WO2008098734A1; Diagnosis and treatment of preeclampsia). SH and BÅ are co-founders and share-holders of Guard Therapeutics formerly named A1M Pharma. This company develops a treatment of acute kidney injury based on α1-microglobulin, but is not involved in clinical development of diagnosis and treatment of preeclampsia, and has not supported this study financially. This does not alter our adherence to PLOS ONE policies on sharing data and materials. The remaining authors declare that the research was conducted in the absence of any commercial or financial relationships that could be construed as a potential conflict of interest.”**

Please know it is PLOS ONE policy for corresponding authors to declare, on behalf of all authors, all potential competing interests for the purposes of transparency. PLOS defines a competing interest as anything that interferes with, or could reasonably be perceived as interfering with, the full and objective presentation, peer review, editorial decision-making, or publication of research or non-research articles submitted to one of the journals. Competing interests can be financial or non-financial, professional, or personal. Competing interests can arise in relationship to an organization or another person. Please follow this link to our website for more details on competing interests: [http://journals.plos.org/plosone/s/competing-interests](about:blank)

3. Thank you for submitting the above manuscript to PLOS ONE. During our internal evaluation of the manuscript, we found some minor text overlap between your Discussion section (limitations) of your submission and the following previously published work:

https://www.ajog.org/article/S0002-9378(19)30909-3/fulltext

Please revise the manuscript to rephrase the duplicated text and ensure you cite your sources.

**Answer: the strengths and limitations section has been modified according to these instructions (lines 430-498).**

4. Please report the source, catalog number, and dilutions of all antibodies used in your study. Please also report the catalog number of all commercial kits and immunoassays used.

**Answer: These information have been added in the methods section (lines 205-259).**

While revising your submission, please upload your figure files to the Preflight Analysis and Conversion Engine (PACE) digital diagnostic tool, [https://pacev2.apexcovantage.com/](https://webmail.clinic.cat/owa/redir.aspx?C=M2diAj363MALIeHVVErxVjoKWpl7-z616W07WHPCX-NHsB2YGh3YCA..&URL=https%3a%2f%2fpacev2.apexcovantage.com%2f). PACE helps ensure that figures meet PLOS requirements. To use PACE, you must first register as a user. Registration is free. Then, login and navigate to the UPLOAD tab, where you will find detailed instructions on how to use the tool. If you encounter any issues or have any questions when using PACE, please email PLOS at [figures@plos.org](https://webmail.clinic.cat/owa/redir.aspx?C=IyUS6z1CMYKymKZ7Rqxbc-7yGRNmmDCwtxs9zHsFdglHsB2YGh3YCA..&URL=mailto%3afigures%40plos.org). Please note that Supporting Information files do not need this step.

**Answer: Thank you for providing this resource. This check has been made.**

**Reviewers' comments:**

**REVIEWER 1**

The study presented by Youssef et al. evaluated maternal and fetal plasma concentrations of heme scavengers (Hemopexin and α1-microglobulin) in different phenotypes of placenta-mediated disorders and controls, both in mothers and neonates.

To prevent the toxic, and notably, the pro-oxidant effect of free hemoglobin and its degradation compounds haem and free iron, the human body disposes of circulating scavenger proteins, including notably those two molecules, that have been associated with preeclampsia.

The study includes 47 singleton pregnancies collected in a monocentric prospective study.

Its originality is related to the exploration of those two haem scavengers both mothers and neonates groups in a same study with the same methods, compared to previously published study.

The manuscript is overall well written and clear. The population is well described, the mention of potential treatments for PE should be cited.

**Answer: The corresponding citation has been added (line 512)**

The limits of the paper are clearly exposed.

The most surprising result is the difference observed for A1 microglobulin between mothers and fetuses, in contrast with hemopexin. This point is debated in the discussion section.

Main corrections

1. A flow chart of the study cohort should be presented.

**Answer: The flow chart of the study has been added to the manuscript as Fig1.**

2. The quality of the figure and legend should be increased. The legend has to permit the reader to understand them without the text. What type of graph is presented? Median IQ? A line showing which populations have been compared with p should be easier to read.

**Answer: The image quality has been increased (currently it’s provided in .eps format). The type of graph has been added to the legend indicating that it is a box plot that represent median and IQ range. A line showing p values has been included in the figure. In addition, the type of graph has also been added to the legend of S3 Fig.**

3. The analysis of the quality of the homemade A1M assay should be presented briefly. Notably, the standard curve should be presented in supporting pieces of information, or a previous publication should be cited, if it do exist. The manufacturer’s providing the monoclonal antibodies should be mentioned. Whether all samples were analyzed in a single batch and if some internal quality controls were included should be precised (since unknown samples are cited).

**Answer: Maternal and fetal A1M average intraassay coefficient of variation were 2.23% (±1.8% standard deviation) and 1.21% (±0.95% standard deviation) respectively. The samples were analyzed in 10 batches (5 maternal and 5 fetal). A new standard curve has been prepared for each plate, an example has been added as S1 Fig with a comment in lines 352-357. The interassay coefficient of variance was assessed in a previous study by Anderson et al. Am J Obstet Gynecol 2011;204:520.e1-5 (it was <4.05%). In the current study, the interassay coefficient of variation has not been assessed. The manufacturer’s providing the monoclonal antibodies has been added in line 358.**

4. As the renal function is supposed to explain the increase of A1M in mothers, an analysis of A1M concentration regarding the creatinine concentration would possibly reinforce this hypothesis. Some additional explanations should be explored, such as albumin and haptoglobin concentrations in maternal and fetal bloods.

**Answer: Thank you for the relevant comment. In fact, we have data concerning creatinine levels in PE mothers since it is measured routinely for clinical purposes, but no data are available for controls, normotensive FGR mothers neither for fetuses from all the study groups. A1M was correlated with creatinine in PE mothers in contrast to no correlation between hemopexin and creatinine. This correlation analysis has been added to the manuscript as S2 Fig. Most unfortunately, Albumin and haptoglobin concentrations were not assessed in the study population. We added this comment to the limitations section line 495.**

Minor corrections

1. The population is extremely well described. However, some data could be suppressed because there are discussed further. For instance, the classification between mild and severe is not use in the analysis of the data, so I’m not sure it deserves to be mentioned in this setting.

**Answer: We agree with the reviewer that some data might be suppressed. The classification to mild and severe preeclampsia has been described in the manuscript to relate it with the sub analysis on preterm and term pregnancies given that the vast majority of preterm cases are severe preeclampsia. However, the criteria of severe preeclampsia have been suppressed with the appropriate reference (line 174).**

2. It should be mentioned whether the assays were done in singlicates for hemopexin.

**Answer: The hemopexin assays were done in duplicates. A comment has been added in the methods section (line 207).**

3. Units have to be corrected ug/mL replace by μg/mL using symbol font.

**Answer: The units have been corrected in both Fig 2 and S3 Fig.**

4. The legend of the supporting tables has to be clarified: I think only mean + Sd is presented for biological values, whereas we can read “Data are mean (Å} standard deviation) or median (interquartile range).”

**Answer: The legend of the supporting tables has been clarified, and “Data are mean (Å} standard deviation) or median (interquartile range)” has been modified into “Data are mean (Å} standard deviation)”.**

5. Line 131 : three instead of 3; Line 132 : two instead of 2.

**Answer: These changes have been made (lines 171-172 in the new version with track changes).**

**REVIEWER 2**

Overall, well performed set of experiments. I have only minor comments.

1. There's obviously a wide range of lab values in each group. Was any attempt made to correlate the levels of A1M or hemopexin with overall outcomes? Time to delivery, maternal morbidity, et cetera.

**Answer: Yes, we have explored the correlation/association between hemopexin or A1M and the pregnancy outcome. None of these two heme scavengers were correlated with time to delivery, maternal morbidity, including severity and days of admission at the intensive care unit, or perinatal morbidity such as low APGAR score at 5 minutes and neonatal intensive care unit admissions. This comment has been added to the manuscript (lines 340-342).**

2. Was any attempt made to measure markers of oxidative stress? Oxidized lipids, for instance?

**Answer: Unfortunately, these markers have not been assessed in the present study. This comment has been added to the limitations section line 496.**

Thank you for your consideration!

Sincerely,

Lina Youssef, MD PhD

Erasmus Mundus Doctorate in Fetal and Perinatal Medicine – FetalMed-PhD
