# Supplementary material for: Hemopexin and α1-microglobulin heme scavengers with differential involvement in preeclampsia and fetal growth restriction
Source: PLoS One. 2020 Sep 11;15(9):e0239030. doi: 10.1371/journal.pone.0239030 (PMC7485876; doi:10.1371/journal.pone.0239030)
Supplement: Attachment — Submitted filename: 200827_Response_to_reviewers.docx [file pone.0239030.s003.docx]

Lina Youssef

Fetal medicine research center, BCNatal

Sabino de Arana 1, 08028 Barcelona, Spain

+34 932 275 400

lyoussef@clinic.cat

August 27, 2020

Dear Editor,

Thank you very much for considering our manuscript “Hemopexin and α1-microglobulin heme scavengers with differential involvement in preeclampsia and fetal growth restriction” (PONE-D-20-14717).

We would like to thank the reviewers for the second round of Reviewers’ comments and the opportunity to further revise the paper: getting the manuscript into the form in which the results are most relevant to and best understood by readers is a high priority for my co-authors and myself, and we appreciate the time and effort the reviewers and editors have invested in assisting with that.

The specific changes made in response to the reviewer comments are detailed below:

**ACADEMIC EDITOR COMMENTS:**

There are some remaining comments that must be addressed by the authors in your revised manuscript.

**Answer: The reviewer comments have been addressed and the mistake has been corrected from “grown” to “growth”.**

**Reviewers' comments:**

**REVIEWER 1**

Thank you for this revised version, that is now for me perfectly acceptable for publication.
I just notice the following mistake to correct : Legend Fig 1 ; FGR, fetal grown restriction : replace by fetal growth restriction

**Answer: The mistake has been corrected in Fig 1 legend as well as Table 1 footnote and other tables in the supporting information.**

Thank you for your consideration!

Sincerely,

Lina Youssef, MD PhD

Erasmus Mundus Doctorate in Fetal and Perinatal Medicine – FetalMed-PhD
